# Long Term Effect of Curcumin in Restoration of Tumour Suppressor p53 and Phase-II Antioxidant Enzymes via Activation of Nrf2 Signalling and Modulation of Inflammation in Prevention of Cancer

**DOI:** 10.1371/journal.pone.0124000

**Published:** 2015-04-10

**Authors:** Laxmidhar Das, Manjula Vinayak

**Affiliations:** Biochemistry and Molecular Biology Laboratory, Department of Zoology (Centre of Advanced Study), Banaras Hindu University, Varanasi-221005, India; Yong Loo Lin School of Medicine, National University of Singapore, SINGAPORE

## Abstract

Inhibition of carcinogenesis may be a consequence of attenuation of oxidative stress via activation of antioxidant defence system, restoration and stabilization of tumour suppressor proteins along with modulation of inflammatory mediators. Previously we have delineated significant role of curcumin during its long term effect in regulation of glycolytic pathway and angiogenesis, which in turn results in prevention of cancer via modulation of stress activated genes. Present study was designed to investigate long term effect of curcumin in regulation of Nrf2 mediated phase-II antioxidant enzymes, tumour suppressor p53 and inflammation under oxidative tumour microenvironment in liver of T-cell lymphoma bearing mice. Inhibition of Nrf2 signalling observed during lymphoma progression, resulted in down regulation of phase II antioxidant enzymes, p53 as well as activation of inflammatory signals. Curcumin potentiated significant increase in Nrf2 activation. It restored activity of phase-II antioxidant enzymes like GST, GR, NQO1, and tumour suppressor p53 level. In addition, curcumin modulated inflammation via upregulation of TGF-β and reciprocal regulation of iNOS and COX2. The study suggests that during long term effect, curcumin leads to prevention of cancer by inducing phase-II antioxidant enzymes via activation of Nrf2 signalling, restoration of tumour suppressor p53 and modulation of inflammatory mediators like iNOS and COX2 in liver of lymphoma bearing mice.

## Introduction

Nuclear factor E2-related factor 2 (Nrf2) is a critical transcription factor that binds to promotor region of a number of genes encoding phase-II antioxidant enzymes [1]. Nrf2 is correlated with routine detoxification process and its increased expression has been described in murine liver, intestine, lung and kidney. There exists high similarity between Nrf2 binding sequence (NF-E2 consensus sequence) and antioxidant response element (ARE), which are regulating elements in promotor region of phase-II antioxidant enzymes like GST and NQO1. GST has protective role against oxidative processes and plays a crucial role in detoxification of various xenobiotics in malignant cells or tissues. GST also exhibits several non catalytic functions like intracellular transport of a wide spectrum of hydrophobic ligands and modulation of signal transduction pathway that leads to sequestering of carcinogens [2]. NQO1 is a cytosolic flavoprotein, expressed constitutively in a wide range of mammalian tissues and cell lines. It catalyzes two-electron reduction of several environmental electrophilic contaminants and endogenous compounds, prevents generation of ROS, scavenges oxygen radicals and hence shows a direct role in protection against oxidative stress. NQO1 regulates stability of p53 in response to oxidative stress [3]. Tumour suppressor p53 restricts abnormal cells by induction of growth arrest/triggering apoptosis. p53 also shows antioxidant property that protects the genome from oxidation by ROS, which is a major cause of DNA damage and genetic instability leading to oncogenic transformation [4, 5]. Nrf2 is critical for maintaining glutathione (GSH) redox state via transcriptional regulation of GR [6]. GSH is a predominant intracellular thiol containing antioxidant in liver. It exhibits versatile functions like regulation of gene expression, apoptosis and antioxidant defence towards modulation of cell proliferation. p53 deficient cancer cells are reported to exhibit reduced induction of Nrf2 target genes as compared to p53 proficient cells, suggesting important role of p53 in activation of Nrf2 in cancer cells [7].

Wild-type p53 and TGF-β are key tumour suppressors which regulate an array of cellular responses. p53 physically interacts with Smads and co-ordinately induces transcription of a number of key tumour suppressive genes [8]. TGF-β acts as an anti proliferative factor at early stages of cancer and regulates cell cycle via downstream target genes involved in driving cellular proliferation [9, 10]. It induces apoptosis in human lymphoma cells and suppresses inflammation [11, 12, 13]. Promotion of inflammation is regulated by COX2 via synthesis of PGE2 in various tissues. Expression of COX2 as well as PGE2 production is regulated by TGF-β1 [14, 15, 16]. COX2 is often co-expressed with iNOS and both are involved in cancer progression by regulating proliferation, apoptosis and angiogenesis etc. iNOS plays a pivotal role in mediation of inflammation via nitric oxide (NO) biosynthesis, which in turn modulates COX2 expression and PGE2 synthesis in inflammatory condition [17]. Moderately increased level of iNOS expression promotes tumour growth, whereas further increase in level is reported to be cytotoxic for tumour cells, thus iNOS shows dual role during tumour development [18]. Liver is reported to possess a peculiar property where tumour cells induce endogenous NO release via upregulation of iNOS, leading to killing of sinusoidal tumour cell and reduced hepatic metastasis [19]. Being a major metabolic and detoxifying organ of the body, liver takes active role in anti oxidative defence system and is greatly affected in advanced cancer. Metastasis and malignancy has been confirmed previously in liver of Dalton’s lymphoma bearing (DL) mice [20].

The use of whole extract of herbal source or single-isolated constituent or a metabolite of an isolated constituent, to achieve desirable health benefits has been an issue of the past several years. Curcumin, a polyphenolic phytochemical is reported to inhibit chemically induced carcinogenesis at multiple organ sites in various animal models. Rapid metabolism and elimination are major factors of low bioavailability of curcumin at tissue levels, irrespective of the route of administration, though metabolites of curcumin remain for longer time in different tissues. However, long term effect of curcumin, after withdrawal of administration is still to be reported. It is not clear whether anti-carcinogenic action of curcumin is due to cumulative effect of its metabolites or due to curcumin itself. Further, it is evidenced that the consumption of whole or partially purified food extracts is more beneficial over single-isolated constituent due to the existence of synergistic interactions among phytochemicals in whole foods [20, 21]. Earlier we have reported that anticarcinogenic action of curcumin during its long term effect is mediated via regulation of glycolytic pathway and angiogenesis, as a result of the consequence of modulation of stress activated genes [20]. Present study is designed to investigate long term effect of curcumin in regulation of Nrf2 mediated phase-II antioxidant enzymes, tumour suppressor p53 and inflammation under oxidative tumour microenvironment in metastatic liver of Dalton’s lymphoma bearing mice.

## Materials and Methods

### Chemicals

All chemicals were of analytical and molecular biology grade as well as endotoxin free, and used without further purification. Curcumin, reagents for RNA isolation, Taq polymerase, PMSF, menadione, Sephadex G50 and HRP conjugated anti-β actin antibody were purchased from Sigma Aldrich. Reverse Transcriptase, Ribonuclease Inhibitor, random primers (hexamers), 100 bp Plus DNA ladder and Klenow enzyme from Fermentase Life Science and TURBO DNA-Free^TM^ Kit I were purchased from Ambion. Gene specific primers for RT-PCR were synthesized from Metabion. Anti-mouse p53 antibody from Imgenix, anti-mouse iNOS and COX2 antibody from Cayman and HRP conjugated goat anti-rabbit secondary antibody from Bangalore Genie. Radiolabelled α^32^P-dCTP from Board of Radiation and Isotope Technology (BRIT) and ECL (Super signal Kit) was purchased from PIERCE Biotechnology HYSEL India Pvt. Ltd.

### Animals and induction T-cell Lymphoma (Dalton’s Lymphoma)

Mice (*Mus musculus*, AKR strain) were bred and maintained under standard laboratory conditions with proper humane care, as per the guidelines of the institutional animal ethical committee, at 25±2°C under 12 h light/12 h dark schedule and provided with standard mice feed, drinking water *ad libitum*. All animal experiments were performed with the approval of Institutional Animal Ethical Committee, Banaras Hindu University. Healthy adult male mice (30±2g, 16–20 week old) were used in the experimental work. Dalton’s lymphoma ascite cells were transplanted in adult male mice through intraperitoneal (i.p.) serial transplantation, as described previously [20]. Dalton’s lymphoma ascite cells were gifted by Prof. Ajit Sodhi, School of Biotechnology, Banaras Hindu University, Varanasi, India. Dalton’s lymphoma is a transplantable non-Hodgkin’s murine T-cell lymphoma, originated in the thymus gland of DBA/2 mouse at National Cancer Institute, Bethesda, M D, in 1947. [22].

Development of DL was confirmed by abdominal swelling and increased body weight, which were visible clearly on 10–11 day post transplantation and DL mice survived for 20±2 days. Growth of Dalton’s lymphoma did not show any major change in body weight and ascite fluid accumulation during first 7–9 days starting from next day of DL transplantation (S1 Fig). Thus first 7–9 days can be compared with the lag-phase/preparatory-phase of sigmoid curve for ascite cell population growth. Therefore, schedule of curcumin treatment to DL mice was selected accordingly for 9 days starting from next day of DL transplantation and mice were sacrificed on day 18 of post DL transplantation (before DL mice dies naturally) to get the long term effect of curcumin. If curcumin is administrated through i.v/i.p, it is metabolised to dihydrocurcumin, tetrahydrocurcumin, hexahydrocurcumin and hexahydrocurcuminol. Curcumin is expected to be completely metabolized to its metabolites during last 9 days after treatment. Hence, the effect should not be due to direct effect of curcumin but might be due to metabolites of curcumin [20].

### Schedule of curcumin treatment to DL mice and tissue collection

Six groups of mice were taken with 6 mice in each group (n = 6) and curcumin treatment to DL mice was scheduled as described previously [20]. All mice were sacrificed on day 18 of post DL transplantation (to avoid septic condition before DL mice die due to tumor) by humanely euthanization (cervical dislocation after anesthetized; mice were exposed to ether presented on a cotton gauze inside a small chamber and ether concentration was approximately 80μl/liter of volume of the container). Liver was excised immediately after sacrifice and washed in chilled normal saline and tissue from each group (n = 6) was pooled and chopped aseptically at 4°C for average result. The tissue was used immediately or preserved at -80°C for further study.

### Electrophoretic mobility shift assay (EMSA)

Nuclear protein was extracted and binding affinity to Nrf2 sequences was determined by electrophoretic mobility shift assay (EMSA) as described previously [23, 24, 25]. Purified synthetic oligonucleotide probes corresponding to NF-E2-consensus sequence (sense 5’-TGGGGAACCTGTGCTGAGTCA-3’, antisense 5’-CTCCAGTGACTCAGCACAGGTTCC-3’ or ARE-consensus sequence (sense 5’-AGTCACAGTGACTCAGCAGAAT-3’, antisense 5’-AGATTCTGCTGAGTCACTGTGA-3’) were annealed, end labelled with [α-32P] CTP using Klenow enzyme. Titration for specificity and binding affinity of synthesized oligonucleotide corresponding to ARE and NFE2 binding element were determined by using unlabelled probe as specific competitor and poly-dI/dC as non-specific competitor respectively. The intensity of complex on autoradiogram was photographed and analyzed by densitometric scanning using Alpha Image Analyser System (Alpha Innotech, San Leandro, CA, U.S.A.).

### RNA isolation and RT-PCR

RNA isolation, cDNA synthesis and amplification were done as described earlier [20, 23, 24, 25]. Expression of Nrf2, isozymes of GST, GR, NQO1, p53, TGF-β1, iNOS and COX2 genes were studied by semi-quantitative RT-PCR using synthesized cDNA. The appropriate primer pairs (S1 Table) were used for PCR reactions using Thermal cycler (Applied Biosystem). Band intensity of amplified products was visualized, photographed and analyzed by using Gel Doc System (Alpha Innotech^EC^) and values were normalized with β-actin as internal control.

### Activity gel assay

Activity of GR and NQO1 as well as activity and isozyme patterns of GST was measured by in gel activity staining. Non-denaturing PAGE analysis of antioxidant enzymes was preferred over immunodetection, because change in activity of an enzyme is associated with metabolic changes and the method utilizes substrate specificity based detection of only active part of enzymes in the same gel. It is considered highly relevant for correlating a change in level of a specific isozyme with that of metabolic alterations at cellular level [20].

#### Glutathione-S-transferase (GST)

The activity gel was performed by non-denaturing PAGE, as per the method of Ricci et al. with minor modification [26]. Equal amount of protein from each sample was separated by 10% non-denaturing PAGE at 4°C. Gel was incubated in 100 mM potassium phosphate buffer (pH-6.5) containing 4.5mM GSH, 1mM CDNB and 1mM NBT at 37°C for 10–15 min under gentle agitation. Then gel was washed in water and transferred to a solution of 100 mM Tris-Cl (pH-9.6) containing 3mM PMS for 3–5 min and illuminated in light till appearance of blue formazen complex bands on the gel. The band intensity of different isozymes were visualized, photographed and analyzed using Gel Doc System (Alpha Innotech^EC^).

#### Glutathione reductase (GR)

The activity staining of GR was performed according to the method of Hou et al. [27]. Equal amount of protein from each sample was separated by 8% non-denaturing PAGE at 4°C. After complete electrophoresis, gel was soaked in 50 mM Tris-Cl (pH-7.9) containing 4 mM GSSG, 1.5 mM NADPH and 2 mM DTNB for 30 min at RT, rinsed with 50 mM Tris-Cl (pH-7.9) and transferred to 1.2 mM NBT and 1.6 mM PMS. GR activity was negatively stained in darkness for 10–15 min at RT with gently shaking and then exposed to light until the appearance of clear zones of GR activity band. The gel was washed in water for destaining and band intensity was visualized, photographed and analyzed using Gel Doc System (Alpha Innotech^EC^).

#### NAD(P)H: quinine oxidoreductase (NQO1)

In-gel activity staining of NQO1 was conducted as described by Wrobel et al. [28]. Equal amount of protein from each sample was separated by 10% non-denaturing PAGE at 4°C. Following electrophoresis, gel was stained in 50 mM Tris-Cl (pH-7.5), 0.3 mg/ml MTT, 1 mM NADH and 30 μM menadione with gentle swirling in dark until colour develops (10–15 min) in the areas having NQO1 activity. Reaction was stopped by transferring the gel to a 5% (v/v) solution of acetic acid. Band intensity was visualized, photographed and analyzed using Gel Doc System (Alpha Innotech^EC^).

### Estimation of redox status

Total glutathione and reduced glutathione were determined as total sulfhydryl (T-SH) content and non-protein sulfhydryl (NP-SH) content respectively using molar absorption coefficient of 13100 M^-1^ cm^-1^, and were expressed in micro moles per mg protein [29]. Redox status was calculated as a ratio of NP-SH to protein-bound sulfhydryl (P-SH) content.

### Western blotting

Equal amount of protein from each sample was separated in 10% SDS-PAGE and transferred to the PVDF membrane overnight at 4°C. Membrane was blocked in 5% non-fat milk in PBS (pH 7.4) for 2h at RT. Blot was incubated overnight at 4°C with rabbit anti-mouse p53 (1:500 dilution, Imgenix) or rabbit anti-mouse iNOS (1:200 dilution, Cayman) or rabbit anti-mouse COX2 (1:500 dilution, Cayman) in 1% BSA and 0.05% Tween-20 in PBS (pH 7.4). Blot was washed and incubated with HRP-conjugated goat anti-rabbit IgG (1:2500 dilution, Bangalore Genei) in PBS (pH 7.4) containing 1% BSA and 0.05% Tween-20 for 2 h at RT. Immunoreactive proteins were detected with ECL super signal kit (Pierce Biotechnology) in X-ray film. Band density values were normalized with β-actin.

### Nitric Oxide (NO) Assay

NO level in liver was estimated by using Griess reagent; 100μl of liver homogenate was mixed with 100μl of Griess reagent (0.1% naphthylethylenediamine dihydrochloride, and 1% sulfanilamide in 5% phosphoric acid), incubated for 10 min at RT and absorbance was measured at 540 nm with a plate reader (ECL ELISA reader). The concentration of NO was determined using a standard curve, generated by taking known quantities of sodium nitrite and values were expressed in terms of μg equivalence of NaNO2/mg protein

### Statistical analysis

Statistical analysis was performed by SPSS software using one-way ANOVA followed by Tucky’s test. Values were expressed as mean ± S.E.M. obtained from three different sets of experiments, p<0.05 was taken as statistically significant (95% confidence interval) compared to N group (#) and DL+DMSO group (*).

## Results

### Effect of curcumin on binding of Nrf2 with ARE consensus sequence

**Fig 1:**
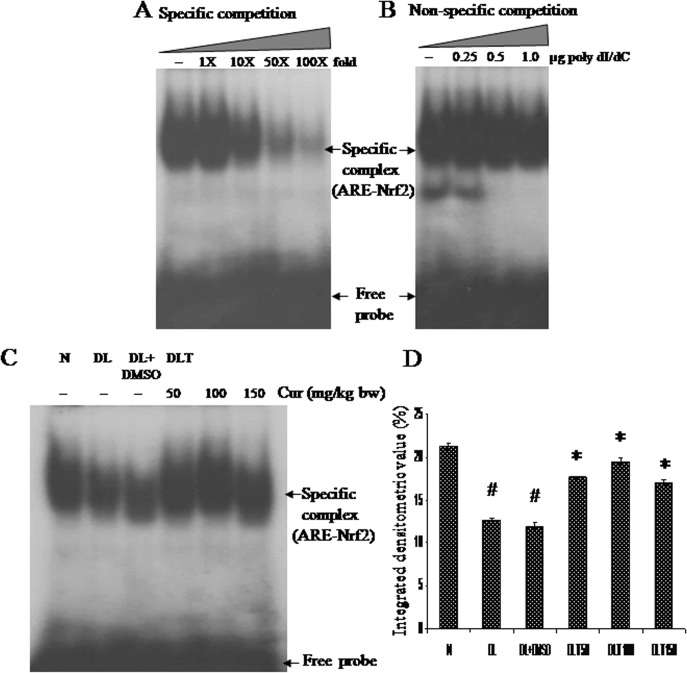
Nrf2 activation (ARE binding). Effect of curcumin on DNA binding activity of Nrf2 with ARE consensus sequences. (A) Titration with unlabelled probe as specific competitor. (B) Titration with poly-dI/dC as non-specific competitor. (C) Autoradiogram showing Nrf2-ARE complex (D) Densitometric scanning of Nrf2-ARE complex. Liver of all six animals of each group was pooled separately and used for extraction of nuclear protein. Data represent mean ± S.E.M. # and * denotes significant difference compared with N and DL+DMSO group respectively. Cur is curcumin and bw is body weight. N, DL, DL+DMSO, DLT50, DLT100 and DLT150 represents normal, Dalton’s lymphoma bearing, Dalton’s lymphoma bearing mice treated with DMSO and Dalton’s lymphoma bearing mice treated with 50, 100 and 150 mg curcumin/kg body weight dissolved in DMSO respectively.

Specificity and binding affinity of synthesized oligonucleotide corresponding to antioxidant response element (ARE consensus sequence) was validated. Titration with unlabelled probe (cold probe) as specific competitor and with non-specific competitor (poly-dI/dC) confirmed specificity and affinity respectively [Fig 1A and 1B]. The intensity of specific DNA-protein complex (ARE-Nrf2) obtained in DL and DL+DMSO mice was approximately 60% and 56% of normal mice respectively. Significant activation and nuclear translocation of Nrf2 was induced by curcumin treatment up to 148%, 164% and 143% of DL+DMSO mice with 50, 100 and 150 mg curcumin/kg bw respectively [Fig 1(C)].

### Effect of curcumin on binding of Nrf2 with NF-E2 consensus sequence

**Fig 2:**
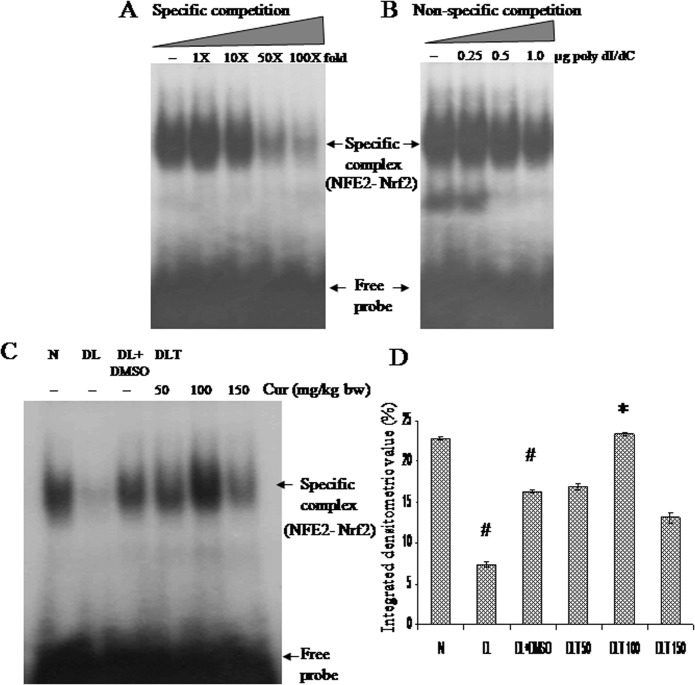
Nrf2 activation (NFE2 binding). Effect of curcumin on DNA binding activity of Nrf2 with NFE2 consensus sequences. (A) Titration with unlabelled probe as specific competitor. (B) Titration with poly-dI/dC as non-specific competitor. (C) Autoradiogram showing Nrf2-NFE2 complex (D) Densitometric scanning of Nrf2-NFE2 complex. Liver of all six animals of each group was pooled separately and used for extraction of nuclear proteins. Data represent mean ± S.E.M. # and * denotes significant difference compared with N and DL+DMSO group respectively. Cur is curcumin and bw is body weight. N, DL, DL+DMSO, DLT50, DLT100 and DLT150 represents normal, Dalton’s lymphoma bearing, Dalton’s lymphoma bearing mice treated with DMSO and Dalton’s lymphoma bearing mice treated with 50, 100 and 150 mg curcumin/kg body weight dissolved in DMSO respectively.

Titration with unlabelled probe as specific competitor and poly-dI/dC as non-specific competitor confirmed specificity and affinity respectively [Fig 2A and 2B]. The intensity of specific DNA-protein complex (NF-E2 with Nrf2) in DL and DL+DMSO mice was approximately 32% and 70% of normal mice respectively. The intensity of the complex was found to be significantly induced with 100 mg curcumin/kg bw which was approximately up to 144% of DL+DMSO mice [Fig 2(C)].

### Effect of curcumin on expression of Nrf2 mRNA

**Fig 3:**
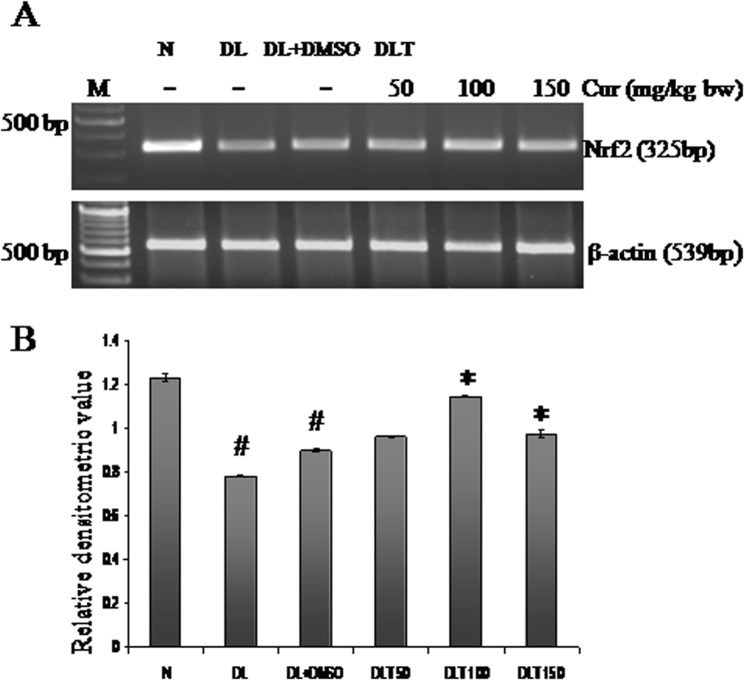
Expression of Nrf2. Effect of curcumin on mRNA expression of Nrf2. (A) RT-PCR of Nrf2 and β-actin. (B) Densitometric scanning of Nrf2 after normalization with β-actin. Liver of all six animals of each group was pooled separately and used for extraction of total RNA. Data represent mean ± S.E.M. # and * denotes significant difference compared with N and DL+DMSO group respectively. Cur is curcumin and bw is body weight. N, DL, DL+DMSO, DLT50, DLT100 and DLT150 represents normal, Dalton’s lymphoma bearing, Dalton’s lymphoma bearing mice treated with DMSO and Dalton’s lymphoma bearing mice treated with 50, 100 and 150 mg curcumin/kg body weight dissolved in DMSO respectively.

The expression of Nrf2 was down regulated in DL and DL+DMSO mice upto approximately 63% and 73% of normal mice respectively, which was significantly up regulated towards normal level by curcumin treatment. The expression of Nrf2 was 107%, 127% and 108% of DL+DMSO with 50, 100 and 150 mg curcumin/kg bw respectively [Fig 3].

### Effect of curcumin on mRNA expression and enzymatic activity of GST isozymes

**Fig 4:**
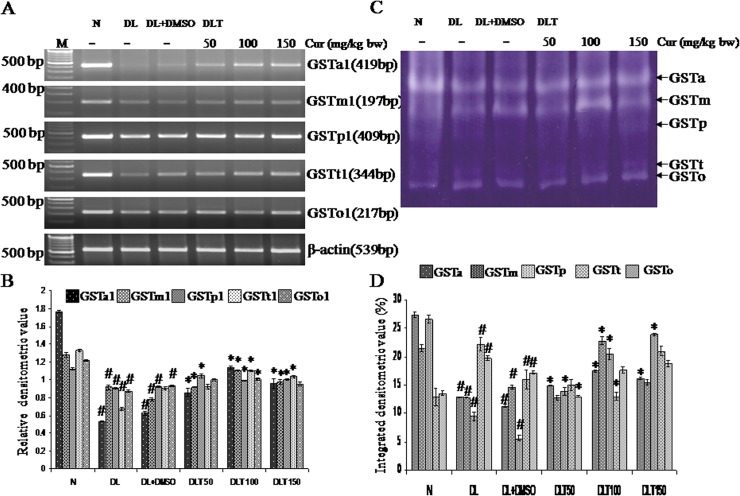
Expression and activities of GST isozymes. Effect of curcumin on expression & enzymatic activity of GST isozymes (A) RT-PCR of GST isozymes and β-actin (B) Densitometric scanning of GST isozymes after normalization with β-actin. (C) Specific staining showing activity of GST isozymes. (D) Densitometric scanning of the activity band of GST isozymes. Liver of all six animals of each group was pooled separately and used for extraction of total RNA and proteins. Data represent mean ± S.E.M. # and * denotes significant difference compared with N and DL+DMSO group respectively. Cur is curcumin and bw is body weight. N, DL, DL+DMSO, DLT50, DLT100 and DLT150 represents normal, Dalton’s lymphoma bearing, Dalton’s lymphoma bearing mice treated with DMSO and Dalton’s lymphoma bearing mice treated with 50, 100 and 150 mg curcumin/kg body weight dissolved in DMSO respectively.

The expression and activity of five isozymes of GST namely GSTa, GSTm, GSTp, GSTt and GSTo were observed in mouse liver, where GSTa is found to be the most abundant isozyme. As compared to normal mice, expression of GSTa, GSTm, GSTp, GSTt and GSTo was down regulated approximately upto 30%, 71%, 80%, 50% and 71% respectively in DL mice and approximately 35%, 61%, 82%, 68%, and 76% respectively in DL+DMSO mice. Curcumin treatment induced expression of five isozymes with 50, 100 and 150 mg curcumin/kg bw as compared to DL+DMSO, which was as follows—GSTa: 137%, 180%, 150%; GSTm: 117%, 110%, 125%; GSTp: 113%, 107%, 108%; GSTt: 102%, 122%, 115%; GSTo: 107%, 108% and 102% respectively [Fig 4(A)].

The activities of five isozymes of GST like GSTa, GSTm, GSTp, GSTt and GSTo did not follow similar variation pattern as that of expression. The activity of GSTa, GSTm and GSTp was reduced in DL and DL+DMSO mice, where as GSTt and GSTo isozymes showed induced activity in DL and DL+DMSO mice as compared to normal mice. The decrease in activity of GSTa: 46%, 41%; GSTm: 59%, 67% and GSTp: 35%, 21% in DL and DL+DMSO mice respectively as compared to normal mice. However, GSTt and GSTo isozymes showed induced activity upto 172% and 123% in case of GSTt and 145% and 126% in case of GSTo respectively in DL and DL+DMSO mice as compared to normal mice. Curcumin modulated activity of isozymes of GST towards normal level. GSTa, GSTm and GSTp were stimulated differentially with different doses of curcumin treatment. All the doses of curcumin elevated the activity of GSTa and GSTp. GSTa was elevated upto approximately 132%, 155%, 143% and GSTp upto approximately 247%, 366%, 420% of DL+DMSO mice with 50, 100 and 150 mg curcumin/kg bw respectively. However, the activity of GSTm was stimulated significantly upto 156%, 106% with 100 and 150 mg curcumin/kg bw respectively. Decrease in activity of GSTt was upto approximately 94%, 81% with 50 and 100 mg curcumin/kg bw respectively and GSTo upto approximately 75% with 50 mg curcumin/kg bw as compared to DL+DMSO mice [Fig 4(C)].

### Effect of curcumin on mRNA expression and enzymatic activity of GR

**Fig 5:**
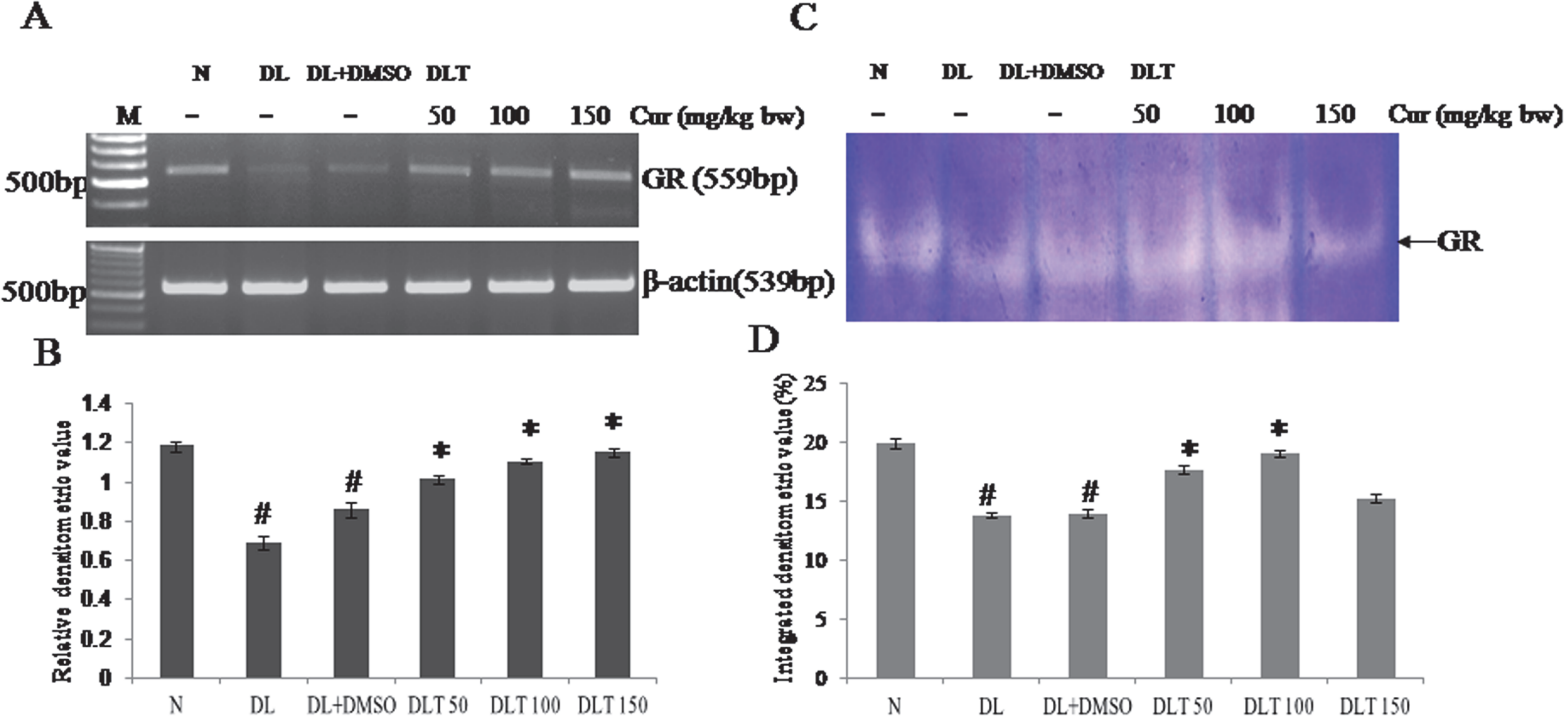
Expression and activity of GR. Effect of curcumin on expression & enzymatic activity of GR (A) RT-PCR of GR and β-actin (B) Densitometric scanning of GR after normalization with β-actin. (C) Specific staining showing activity of GR. (D) Densitometric scanning of the activity band of GR. Liver of all six animals of each group was pooled separately and used for extraction of total RNA and proteins. Data represent mean ± S.E.M. # and * denotes significant difference compared with N and DL+DMSO group respectively. Cur is curcumin and bw is body weight. N, DL, DL+DMSO, DLT50, DLT100 and DLT150 represents normal, Dalton’s lymphoma bearing, Dalton’s lymphoma bearing mice treated with DMSO and Dalton’s lymphoma bearing mice treated with 50, 100 and 150 mg curcumin/kg body weight dissolved in DMSO respectively.

The expression of GR in liver of DL and DL+DMSO mice was down regulated upto approximately 58% and 72% of normal mice respectively. Curcumin treatment induced expression of GR in a dose dependent manner. Up regulation of expression of GR by curcumin is approximately 118%, 129%, 134% of DL+DMSO mice with 50, 100 and 150 mg curcumin/kg bw respectively [Fig 5A and 5B]. The activity of GR was found to be decreased in DL and DL+DMSO mice upto approximately 69% and 70% of normal mice. Treatment of curcumin significantly elevated the activity of GR upto approximately 126%, 136%, 109% of DL+DMSO mice with 50, 100 and 150 mg curcumin/kg bw respectively [Fig 5C and 5D].

### Redox status

**Fig 6:**
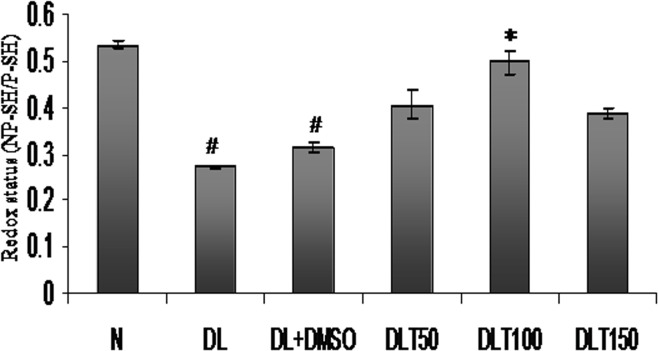
Cellular redox status. Effect of curcumin on cellular redox status in terms of NP-SH/P-SH. Liver of all six animals of each group was pooled separately and used for extraction of total proteins. Data represent mean ± S.E.M. # and * denotes significant difference compared with N and DL+DMSO group respectively. Cur is curcumin and bw is body weight. N, DL, DL+DMSO, DLT50, DLT100 and DLT150 represents normal, Dalton’s lymphoma bearing, Dalton’s lymphoma bearing mice treated with DMSO and Dalton’s lymphoma bearing mice treated with 50, 100 and 150 mg curcumin/kg body weight dissolved in DMSO respectively.

Redox status in liver of lymphoma bearing mice was measured in terms of reduced versus oxidized form of glutathione (NP-SH/P-SH), as redox status is also an important indicator of oxidative stress. The status was observed to be 51% and 58% of normal mice in DL and DL+DMSO mice respectively. All the three doses elevated redox status significantly, which was upto approximately 131%, 159% and 124% of DL+DMSO mice with 50, 100 and 150 mg curcumin/kg bw respectively [Fig 6].

### Effect of curcumin on mRNA expression and enzymatic activity of NQO1

**Fig 7:**
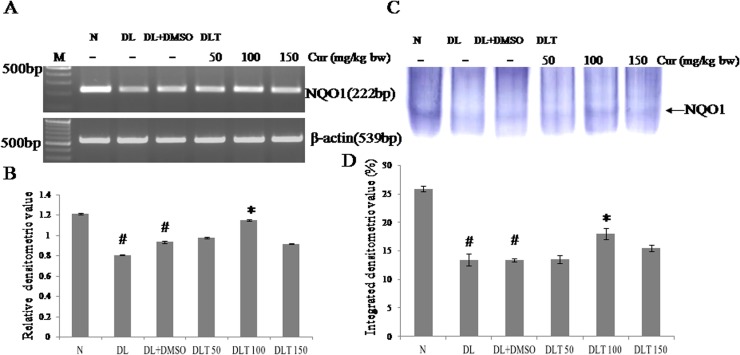
Expression and activity of NQO1. Effect of curcumin on mRNA expression & enzymatic activity of NQO1 (A) RT-PCR of NQO1 and β-actin (B) Densitometric scanning of NQO1 mRNA after normalization with β-actin. (C) Specific staining showing activity of NQO1. (D) Densitometric scanning of the activity band of NQO1. Liver of all six animals of each group was pooled separately and used for extraction of total RNA and proteins. Data represent mean ± S.E.M. # and * denotes significant difference compared with N and DL+DMSO group respectively. Cur is curcumin and bw is body weight. N, DL, DL+DMSO, DLT50, DLT100 and DLT150 represents normal, Dalton’s lymphoma bearing, Dalton’s lymphoma bearing mice treated with DMSO and Dalton’s lymphoma bearing mice treated with 50, 100 and 150 mg curcumin/kg body weight dissolved in DMSO respectively.

Lymphoma bearing mice showed reduced expression of phase II antioxidant enzyme NQO1. The expression was down regulated in DL and DL+DMSO mice upto approximately 66% and 77% of normal mice. Curcumin treatment significantly up regulated expression towards normal level (95% of normal) with the dose of 100 mg curcumin/kg bw. The increased expression of NQO1 by curcumin was approximately 104% and 123% of DL+DMSO mice with 50 and 100 mg curcumin/kg bw respectively [Fig 7A and 7B]. The activity of NQO1, observed by activity gel assay follows the variation pattern of its expression. It was found to be decreased in DL and DL+DMSO mice upto approximately 52% and 51% of normal mice respectively. Curcumin treatment with 100 and 150 mg/kg bw elevated the activity of NQO1 significantly, which was approximately upto 134% and 115% of DL+DMSO mice respectively [Fig 7C and 7D].

### Effect of curcumin on mRNA expression and protein level of p53

**Fig 8:**
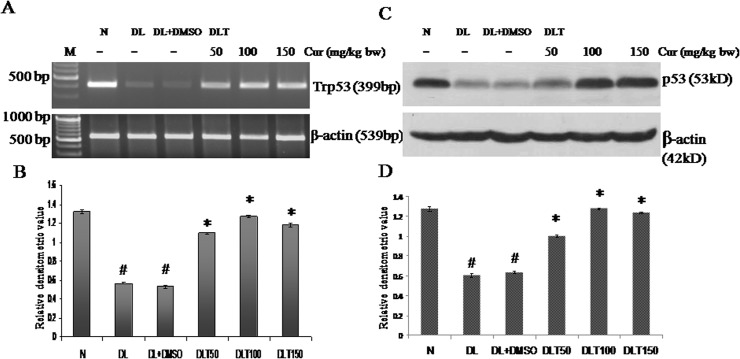
Expression of p53. Effect of curcumin on mRNA expression and protein level of p53 (A) RT-PCR of p53 and β-actin (B) Densitometric scanning of p53 mRNA, after normalization with β-actin. (C) Western analysis of p53 and β-actin. (D) Densitometric scanning of p53 after normalization with β-actin. Liver of all six animals of each group was pooled separately and used for extraction of total RNA and proteins. Data represent mean ± S.E.M. # and * denotes significant difference compared with N and DL+DMSO group respectively. Cur is curcumin and bw is body weight. N, DL, DL+DMSO, DLT50, DLT100 and DLT150 represents normal, Dalton’s lymphoma bearing, Dalton’s lymphoma bearing mice treated with DMSO and Dalton’s lymphoma bearing mice treated with 50, 100 and 150 mg curcumin/kg body weight dissolved in DMSO respectively.

The p53 being a labile protein, is degraded fast under oxidative stress. The mRNA expression of Trp53 (p53 gene) was found to be down regulated in DL and DL+DMSO mice upto approximately 43% and 40% of normal mice. All the three doses of curcumin induced expression of Trp53 significantly. The up regulation of Trp53 expression was approximately upto 200%, 240% and 223% of DL+DMSO mice with 50, 100 and 150 mg curcumin/kg bw respectively [Fig 8A and 8B]. The level of tumour suppressor p53 protein follows similar variation pattern of its expression. Protein level was decreased in case of DL and DL+DMSO mice upto approximately 47% and 50% of normal mice respectively. Curcumin treatment resulted in significant enhancement of level of p53, which was approximately 158%, 200% and 195% of DL+DMSO mice with 50, 100 and 150 mg curcumin/kg bw respectively [Fig 8C and 8D].

### Effect of curcumin on mRNA expression and protein level of iNOS

**Fig 9:**
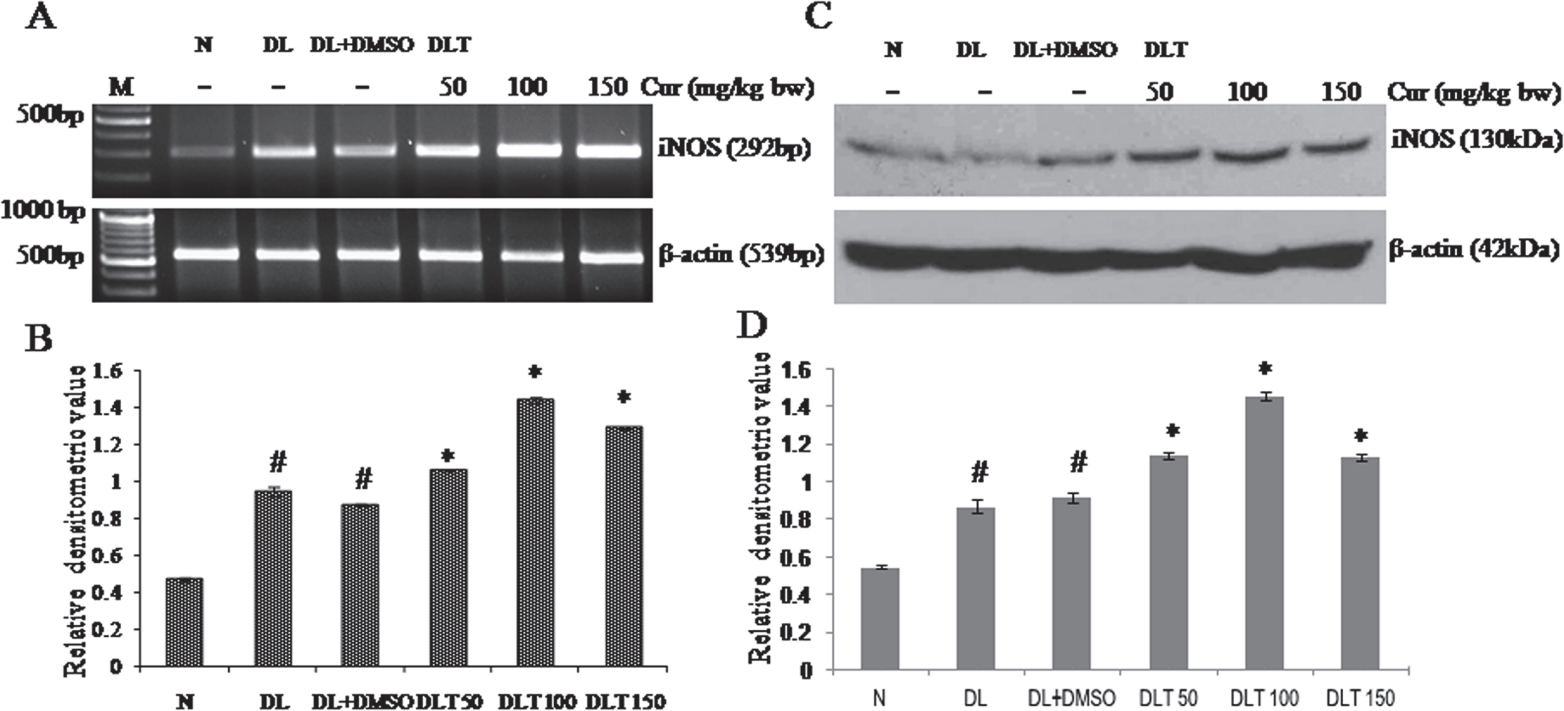
Expression of iNOS. Effect of curcumin on mRNA expression and protein level of iNOS (A) RT-PCR of iNOS and β-actin (B) Densitometric scanning of iNOS mRNA after normalization with β-actin. (C) Western analysis of iNOS and β-actin (D) Densitometric scanning of iNOS after normalization with β-actin. Liver of all six animals of each group was pooled separately and used for extraction of total RNA and proteins. Data represent mean ± S.E.M. # and * denotes significant difference compared with N and DL+DMSO group respectively. Cur is curcumin and bw is body weight. N, DL, DL+DMSO, DLT50, DLT100 and DLT150 represents normal, Dalton’s lymphoma bearing, Dalton’s lymphoma bearing mice treated with DMSO and Dalton’s lymphoma bearing mice treated with 50, 100 and 150 mg curcumin/kg body weight dissolved in DMSO respectively.

The expression of iNOS mRNA was observed to be up regulated upto 2.03-fold and 1.86-fold in DL and DL+DMSO mice respectively, as compared to normal mice. All the doses of curcumin significantly induced the expression of iNOS, which was approximately upto 122%, 166% and 148% of DL+DMSO mice with 50, 100 and 150mg/kg bw respectively [Fig 9A and 9B]. Protein level of iNOS in liver of DL and DL+DMSO mice was found to be upregulated approximately upto 1.6-fold and 1.7-fold respectively compared to normal mice. Following the pattern of mRNA expression, protein level of iNOS was elevated by curcumin treatment approximately upto 125%, 160% and 123% of DL+DMSO mice with the doses of 50, 100 and 150 mg/kg bw respectively [Fig 9C and 9D].

### Effect of curcumin on NO level

**Fig 10:**
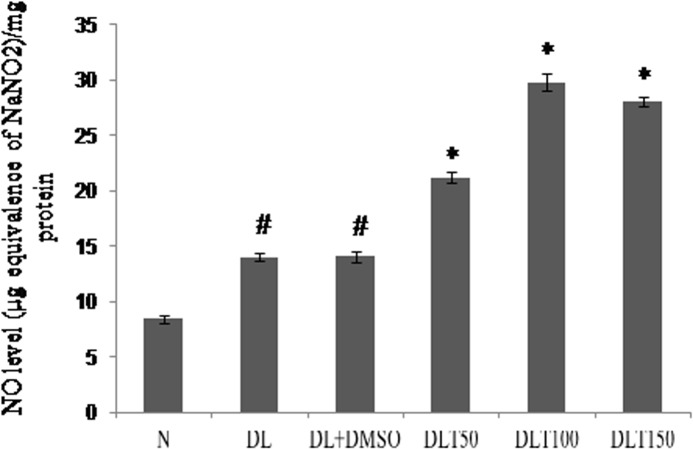
Cellular NO level. Effect of curcumin on NO level in liver tissue. NO level was observed by using Griess reagent and expressed in μg equivalence of NaNO2/mg protein. Liver of all six animals of each group was pooled separately and used for extraction of total proteins. Data represent mean ± S.E.M. # and * denotes significant difference compared with N and DL+DMSO group respectively. Cur is curcumin and bw is body weight. N, DL, DL+DMSO, DLT50, DLT100 and DLT150 represents normal, Dalton’s lymphoma bearing, Dalton’s lymphoma bearing mice treated with DMSO and Dalton’s lymphoma bearing mice treated with 50, 100 and 150 mg curcumin/kg body weight dissolved in DMSO respectively.

Activity of iNOS was measured in terms of NO level, as iNOS is the major contributor of NO production in liver. In comparison to normal mice, NO concentration in DL and DL+DMSO mice was observed to be 165% and 166% higher respectively. All the doses of curcumin treatment further induced the production of NO level significantly upto 150%, 211% and 198% of DL+DMSO mice with the dose of 50, 100 and 150mg/kg bw respectively [Fig 10].

### Effect of curcumin on mRNA expression of TGF-β1

**Fig 11:**
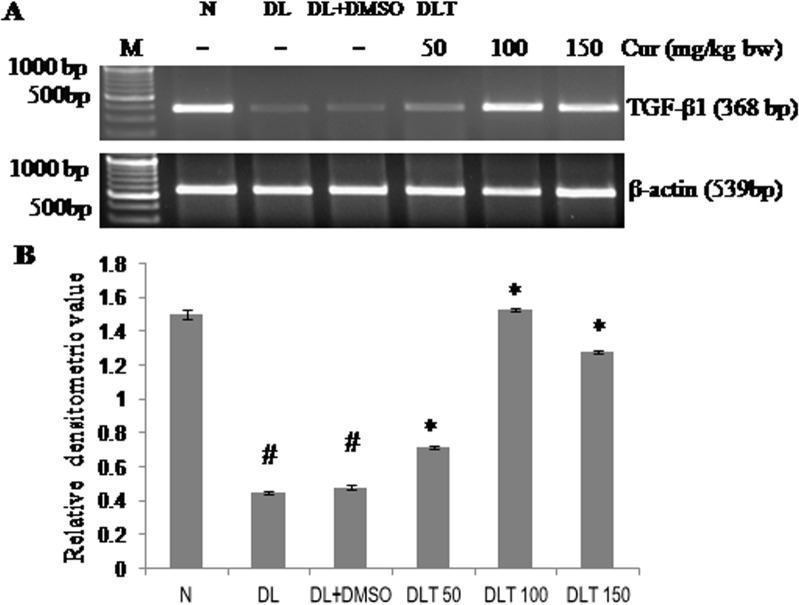
Expression of TGF-β1. Effect of curcumin on mRNA expression of TGF-β1 (A) RT-PCR of TGF-β1 and β-actin (B) Densitometric scanning of TGF-β1 after normalization with β-actin. Liver of all six animals of each group was pooled separately and used for extraction of total RNA. Data represent mean ± S.E.M. # and * denotes significant difference compared with N and DL+DMSO group respectively. Cur is curcumin and bw is body weight. N, DL, DL+DMSO, DLT50, DLT100 and DLT150 represents normal, Dalton’s lymphoma bearing, Dalton’s lymphoma bearing mice treated with DMSO and Dalton’s lymphoma bearing mice treated with 50, 100 and 150 mg curcumin/kg body weight dissolved in DMSO respectively.

Expression of TGF-β1 mRNA was found to be down regulated approximately upto 29% and 32% in DL and DL+DMSO mice respectively as compared to normal mice. Curcumin modulated expression of TGF-β1 mRNA towards normal level. TGF-β1 mRNA expression was elevated approximately upto 150%, 300% and 265% of DL+DMSO mice with the dose of 50, 100 and 150mg/kg bw respectively [Fig 11].

### Effect of curcumin on mRNA expression and protein level of COX2

**Fig 12:**
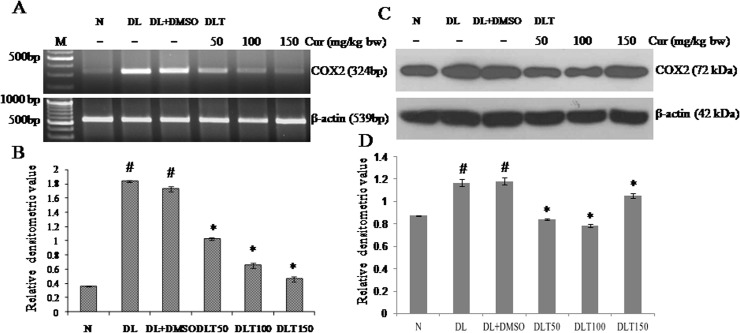
Expression of COX2. Effect of curcumin on mRNA expression and protein level of COX2 (A) RT-PCR of COX2 and β-actin (B) Densitometric scanning of COX2 mRNA after normalization with β-actin. (C) Western analysis of COX2 and β-actin (D) Densitometric scanning of COX2 after normalization with β-actin. Liver of all six animals of each group was pooled separately and used for extraction of total RNA and proteins. Data represent mean ± S.E.M. # and * denotes significant difference compared with N and DL+DMSO group respectively. Cur is curcumin and bw is body weight. N, DL, DL+DMSO, DLT50, DLT100 and DLT150 represents normal, Dalton’s lymphoma bearing, Dalton’s lymphoma bearing mice treated with DMSO and Dalton’s lymphoma bearing mice treated with 50, 100 and 150 mg curcumin/kg body weight dissolved in DMSO respectively.

DL mice showed higher level of COX2 expression. It was upregulated in DL and DL+DMSO mice upto approximately 508% and 478% of normal mice respectively. Curcumin treatment modulated it towards normal level by down regulating the expression in a dose dependent manner, which was approximately 59%, 38% and 27% of DL+DMSO mice with 50, 100 and 150 mg curcumin/kg bw respectively [Fig 12A and 12B]. The variation pattern of protein level of COX2 in DL and DL+DMSO mice was approximately 133% and 135% of normal mice respectively. Curcumin treatment significantly decreased the protein level of COX2, which was approximately 71%, 66% and 88% of DL+DMSO mice with 50, 100 and 150mg curcumin/kg bw respectively [Fig 12C and 12D].

## Discussion

Being a vital organ, involvement of liver is a major determinant of survival from cancer. Most of the patients die due to hepatic metastases, irrespective of the origin of cancer; although true prevalence of metastatic liver disease is yet to be studied. Metastatic cancer cells trigger a proinflammatory response and reactive oxygen intermediates released by cancer cells in hepatic sinusoid microenvironment, leading to development of oxidative tumour microenvironment [20].

Our earlier findings show induced activity of NF-κB under oxidative tumour microenvironment in liver of DL mice, which is correlated with impaired antioxidant defence system and higher level of proinflammatory cytokines [23, 24, 25]. This may lead disturbed cross-talks between NF-κB and Nrf2 pathway, as Nrf2 regulates cellular antioxidant defence system by modulating its downstream target genes of phase II detoxifying enzymes like NQO1 and GST [30, 31].

Therefore, we evaluated Nrf2 signalling in DL mice. Our finding of reduced Nrf2 signalling is correlated with high oxidative stress in DL mice in terms of redox status. Elevated binding of nuclear Nrf2 to ARE and NFE2 by curcumin treatment is capable of restoring Nrf2 expression with modulation of redox status towards normal level. Expression of GST and NQO1 are reported to be decreased abruptly in Nrf2 knockout mice as compared to wild-type counterpart [32]. Nrf2 in association with other transcription factors like c-Jun and small Maf, plays an important role in preventing carcinogenesis in liver via ARE-mediated induction of phase-II detoxifying enzymes [33]. Further, the stability of Nrf2 protein is more important than its mRNA expression and protein synthesis. Significant increase in expression and nuclear translocation of Nrf2 by curcumin treatment suggests that stabilization of Nrf2 protein might be involved in curcumin-induced accumulation of nuclear Nrf2.

Long term effect of curcumin is likely to inhibit the ability of Cullin3-Rbx1 E3 ubiquitin ligase to target Nrf2, needed for its ubiquitinization, facilitating activation and nuclear translocation of Nrf2 [34]. The existence of ARE sequences in the promotor region of GST genes renders regulation of their expression via Nrf2. Enhanced cellular detoxification system by Nrf2 via GST is reported to contribute towards cancer chemoprevention [35]. The isozymes of mammalian GST super family are expressed in a tissue-specific manner [36]. Different isozymes of GST are activated antagonistically during regulation of cancer. GSTa, GSTm and GSTp are known to have tumour preventing role. Reduced expression of GSTa has been linked to an increased risk in cancer. GSTm null phenotype is associated with an increased risk of cancer development in lung, colon and bladder [37]. GSTp acts as an endogenous inhibitor of JNK1 via protein-protein interactions, and hence involved in stress response and cell proliferation. Low activity of GSTp favors activation of MAP kinase pathway owing to oxidative stress [38]. These reports support our finding of low activity of these isozymes in DL mice. Anti-carcinogenic property of curcumin is exhibited by elevating the activities of GSTa, GSTm and GSTp. In contrast to GSTa, GSTm and GSTp, higher activity of GSTt and GSTo are linked to increased susceptibility to cancer and altered cellular redox conditions [39]. Higher activity of these two isozymes in DL mice confirmed their pro-carcinogenic action. Curcumin treatment to DL mice significantly decreased activity of tumour promoting isozymes GSTt and GSTo showing its anti-carcinogenic potential. Activity of GST is redox sensitive as it requires GSH. Significantly low redox status in liver of DL mice as compared to normal mice coincides with variation in activity of Nrf2. Thus, curcumin might have improved redox status of DL mice towards normal level via Nrf2.

Further, Nrf2 plays an essential role in regulation of nonprotein thiol GSH level in oxidative tumour microenvironment through antioxidant genes [40]. Upregulation of GSH levels by curcumin in liver of lymphoma bearing mice maintains intracellular redox balance and protects against oxidative insult. In addition, GSH can detoxify carcinogens and plays an important role in free radical scavenging [35]. Lower activity of GR under oxidative microenvironment favours accumulation of GSSG in liver of DL mice. The deficiency of GR is characterized by increased sensitivity of membranes to H_2_O_2_ and contributes to oxidative stress, which plays a key role in pathogenesis of many diseases including cancer [41]. The findings suggest that curcumin reduced oxidative stress in liver of DL mice by inducing expression and activity of GR via Nrf2 signalling, which enhances GSH level and there by activates GST.

Reduced oxidative stress in curcumin treated mice might be due to superoxide scavenging role of NQO1. Further, NQO1 regulates stability of p53 by inhibiting its ubiquitin-independent 20S proteasomal degradation [42]. NQO1 deficient mice show reduced p53 induction and apoptosis, impaired NF-κB function and increased susceptibility to cancer progression [43].

Curcumin is reported to inhibit activity of NQO1 *in vitro*, which may be due to change in specific conformation of NQO1 at Tyr 128 and Phe 232 by direct interaction with curcumin, that is important for interaction with p53 [44, 45]. However, in case of animal system such finding is not yet reported. Loss of p53 is required for tumour development and maintenance as evidenced by p53 knockout mice. Certain tumours do not express p53 and in some cases reduced transcription of the p53 gene observed [46]. Hence, p53 restoration may be a promising therapeutic strategy. However, stability of p53 is an important factor over its expression. NQO1-mediated p53 stabilization is especially prominent under induction of oxidative stress. NQO1 can stabilize endogenous as well as transfected wild-type p53 [47]. Low p53 level in DL mice coincides with decreased expression and activity of NQO1 and its improvement by curcumin restores the level of p53. Antioxidant role of p53 further contributes to suppression of oxidative stress in liver of DL mice. A number of proteins involved in protection against oxidative stress are up regulated by p53 [3]. p53 is a suppressor of NF-κB mediated inflammatory response [48]. Earlier we have reported that curcumin inhibits NF-κB activity via activation of antioxidant defence system, contributing to its anti-carcinogenic action [23]. Thus, restoration of level as well as function of p53 by curcumin correlates with regression of lymphoma in DL mice. Mechanism responsible for tumour regression is dependent on tumour type. Consequence of p53 restoration is correlated with induction of apoptosis in lymphomas [49]. Our result supports the mechanism of p53 action in tumour regression via suppression of cell proliferation. Tumour regression has been observed in terms of body weight, ascite fluid accumulation, proliferation and viability of ascite cells [23]. NQO1 thus plays a dual role in protection against carcinogenesis. Its antioxidant properties protect the cell from carcinogenic oxidative damage, and its ability to stabilize p53 supports elimination of cancer prone cells [50].

Modulation of level of NO production by tumour cells at the site of metastasis could be crucial [51]. Both promoting and deterring actions of NO have been reported, probably depending upon the local concentration of NO within tumour microenvironment. NO shows proinflammatory activity at the range of 100–300 nM, however more than 500 nM concentration shows anti-inflammatory activity [52]. Higher NO production by NO donor drugs or gene therapy with iNOS has been shown to inhibit tumour growth [53, 54]. Pivotal effects in liver cells such as malignant transformation, angiogenesis and metastasis are modulated by iNOS. On the other hand, NO derived from macrophages has a potentially cytotoxic or cytostatic effect on tumour cells [55]. Upregulation of iNOS at transcriptional level as well as at translational level in DL mice suggests its proinflammatory activity and its role in metastasis and inflammation. Induced expression and level of iNOS by curcumin activates NO production which in turn promotes its anti-inflammatory action [56].

Tumour progression is regulated by TGF-β via simultaneous activation of Smad-mediated mitoinhibition and decreased PGE2 production [57]. However, function of TGF-β in cancer depends on the stage and context of tumour. TGF-β shows anti-tumoural activity at early stages by inhibiting proliferation of tumour cells, whereas expression of TGF-β is suppressed or its signalling pathways are inactivated at later stages [51, 58, 59]. Thus, our result of reduced expression of TGF-β1 in DL mice suggests inflammatory tumour microenvironment owing to hepatic metastasis. Upregulation of expression of TGF-β1 towards normal level by curcumin suggests its anti carcinogenic effect. Besides, TGF-β1 elicits Nrf2-mediated antioxidant responses in aortic smooth muscle cells and in airway smooth muscle cells, which supports our finding of increased expression of TGF-β1, Nrf2 as well as iNOS level following curcumin treatment [56, 60, 61]. The action of TGF-β1 and COX2 are antagonistic [16]. Reduced expression of TGF-β1 found in DL mice supports up regulation of COX2, as stimuli like oxidative stress and secretion of proinflammatory cytokines in tumour microenvironment of DL liver induce expression of COX2 [62]. This is further supported by our earlier finding of NF-kB activation [23]. Thus, deregulation in expression of oncogenes, tumour-suppressor genes and stability genes are involved in carcinogenesis by reducing level of antioxidant defence in tumour microenvironment which induces inflammation and carcinogenesis [20].

Thus, curcumin restored phase-II antioxidant enzyme activities and tumour suppressor p53 via activation of Nrf2 signalling which leads to upregulation of TGF-β and inhibition of carcinogenesis via reciprocal regulation of iNOS and COX2 in liver of lymphoma bearing mice.

Low bioavailability of curcumin at tissue level is due to rapid metabolism and elimination, irrespective of the route of administration. However, metabolites of curcumin remain for longer time in different tissues. Therefore the effect of curcumin, even after withdrawal of treatment might be due to synergistic action of its various metabolites. However, this needs further verification using different isolated metabolites. The treatment with 100 mg curcumin/kg body weight was found to be optimal dose. 50 mg/kg body weight may be insufficient to produce the maximum effect and 150 mg/kg body weight may lead to negative feedback regulation.

In summary, long term effect of curcumin potentiates its tumour preventing action by inducing phase-II antioxidant enzymes via activation of Nrf2 signalling, restoration of tumour suppressor p53 and modulation of inflammatory mediators like TGF-β and COX2 in liver of lymphoma bearing mice. Thus the observations suggest antioxidant and anti-inflammatory property of the metabolites of curcumin.

## Supporting Information

S1 Fig Growth pattern/change in body weight of DL mice. Body weights of DL mice were measured every day, starting from one day after DL transplantation till 18^th^ day, to observe the growth pattern of Dalton’s lymphoma. Body weight verses time duration curve follows sigmoid curve of cell population growth (here ascite cell population growth).
(TIF)

S1 Table Primer pairs and conditions of PCR. Primer pairs of corresponding gene with annealing and elongation condition as well as optimum cycle for amplification.
(PDF)
