# Supplementary figures and images for: Long Term Effect of Curcumin in Restoration of Tumour Suppressor p53 and Phase-II Antioxidant Enzymes via Activation of Nrf2 Signalling and Modulation of Inflammation in Prevention of Cancer
Source: PLoS One. 2015 Apr 10;10(4):e0124000. doi: 10.1371/journal.pone.0124000 (PMC4393109; doi:10.1371/journal.pone.0124000)

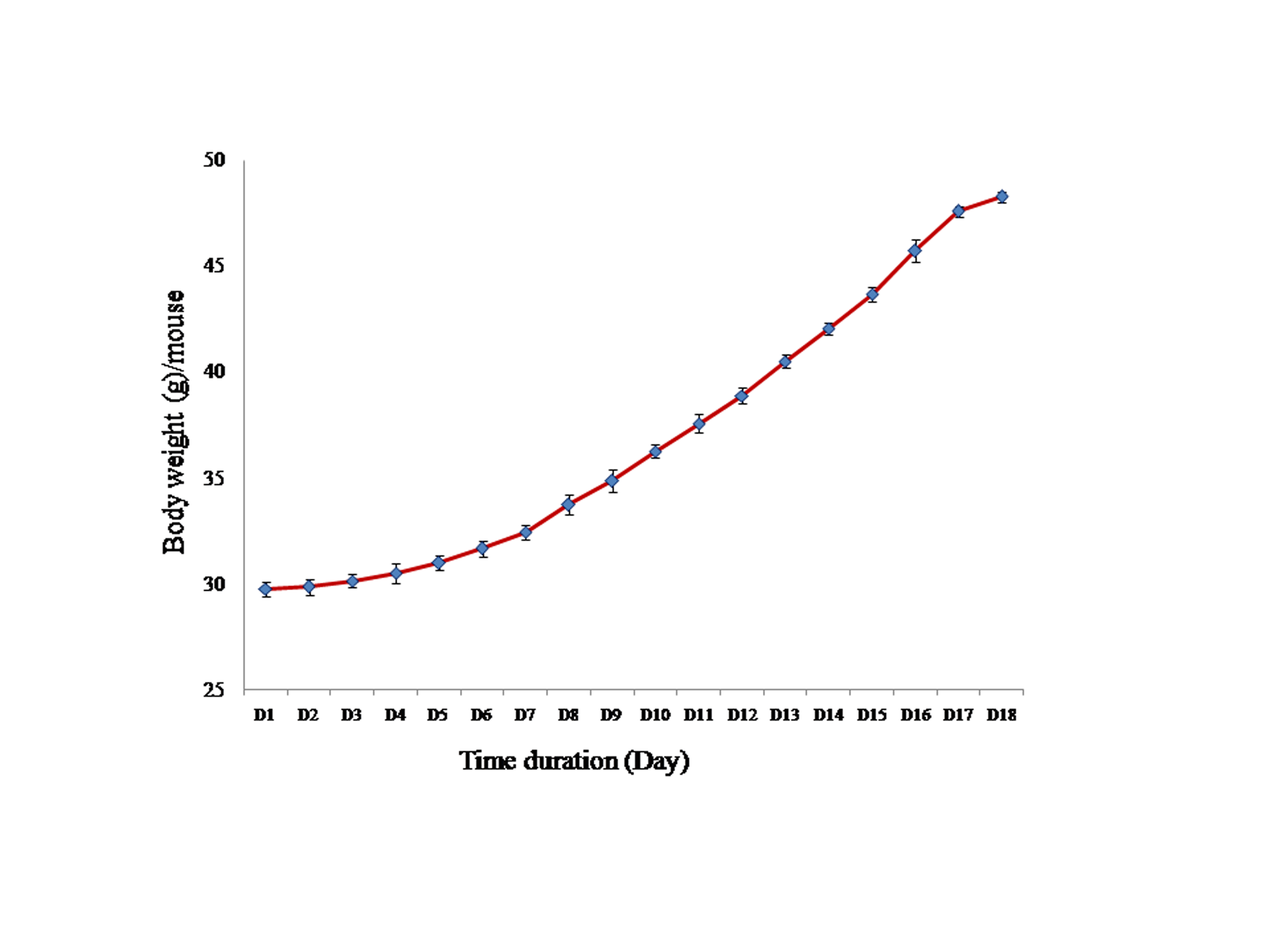

Supplement: S1 Fig — Body weights of DL mice were measured every day, starting from one day after DL transplantation till 18th day, to observe the growth pattern of Dalton’s lymphoma. Body weight verses time duration curve follows sigmoid curve of cell population growth (here ascite cell population growth). (TIF) [file pone.0124000.s001.tif]
