# Supplementary material for: Long Term Effect of Curcumin in Restoration of Tumour Suppressor p53 and Phase-II Antioxidant Enzymes via Activation of Nrf2 Signalling and Modulation of Inflammation in Prevention of Cancer
Source: PLoS One. 2015 Apr 10;10(4):e0124000. doi: 10.1371/journal.pone.0124000 (PMC4393109; doi:10.1371/journal.pone.0124000)
Supplement: S1 Table — Primer pairs of corresponding gene with annealing and elongation condition as well as optimum cycle for amplification. (PDF) [file pone.0124000.s002.pdf]

Genes with accession no.	Sequences of primer pairs (F: forward, R: reverse)	PCR condition (annealing and elongation)	No. of cycles	Amplicon size (bp)
Nrf2 (NM_010902.3)	F: 5’-TGCTGCAAGTAGCCTCGCCG-3’<br>R: 5’-TGCTGGGCCGGCTGAATTGG-3’	65°C-40sec<br>72°C-40sec	24	325
GSTa1 (NM_008181.3)	F: 5’-AAGCTGGCGCAGACCAGAGC-3’<br>R: 5’-TGGCTGCCAGGCTGTAGGAACT-3’	65°C-45sec<br>72°C-45sec	26	419
GSTm1 (NM_010358.5)	F: 5’-TGGGATTGGTGCAGGGTTGGG-3’<br>R: 5’-AGCATGCGGATCGGGTGTGTC-3’	59°C-30sec<br>72°C-30sec	26	197
GSTp1 (NM_013541.1)	F: 5’-TGTCTACGCAGCACTGAATCCGC-3’<br>R: 5’-CCCAGGCAGGGCCTTCACGTA-3’	64°C-45sec<br>72°C-45sec	24	409
GSTt1 (NM_008185.3)	F: 5’-ACAGAAGTGGTTCCCGCCGC-3’,<br>R: 5’-AGGCCCGTATGCTGCCATGC-3’	59°C-30sec<br>72°C-30sec	22	344
GSTo1 (NM_010362.2)	F: 5’-GTGCTTCCAGAGTCGCCCGC-3’<br>R: 5’-CGTGCCGGATTCCCTTGGCC-3’	64°C-45sec<br>72°C-45sec	27	217
GR (NM_010344.4)	F: 5’-CTTCCTTCGACTACCTGG-3’<br>R: 5’-ATGCCTGCGATCTCCACA-3’	55°C-30sec<br>72°C-30sec	36	559
NQO1 (NM_008706.5)	F: 5’-TCACCACTGGGGGTAGCGGC-3',<br>R: 5’-GTGGGGTCTCCTCCCAGACGG-3'	63°C-30sec<br>72°C-40sec	32	222
Trp53 (NM_011640.3)	F: 5’-CGGAGGTCGTGAGACGCTGC-3'<br>R: 5'-TCCCTGGGGGCAGTTCAGGG-3'	62°C-45sec<br>72°C-45sec	26	399
TGF-β1 (NM_011577.1)	F: 5’-AGCCCGAAGCGGACTACTAT-3’<br>R: 5’-GCTGAATCGAAAGCCCTGTA-3’	58°C/45sec<br>72°C/45sec	28	368
iNOS (NM_010927.3)	F: 5’- GGATGCCTTCCGCAGCTGGG -3’<br>R: 5’- TCGGCTGCCCTCGAAGGTGA -3’	64°C/45sec<br>72°C/45sec	27	292
COX2 (NM_011198.3)	F: 5’- TTGAAGACCAGGAGTACAGC -3’<br>R: 5’- GGTACAGTTCCATGACATCG -3’	54°C/40sec<br>72°C/40sec	30	324
β-actin (NM_007393.3)	F: 5’-GTGGGCCGCCCTAGGCACCAG-3’<br>R: 5’-TCTTTGATGTCACGCACGATTTC-3’	60°C/45sec<br>72°C/45sec	26	539
